# Long Axial Field-of-View PET/CT Could Answer Unmet Needs in Gynecological Cancers

**DOI:** 10.3390/cancers15092407

**Published:** 2023-04-22

**Authors:** Elizabeth Katherine Anna Triumbari, Vittoria Rufini, Clemens Mingels, Axel Rominger, Abass Alavi, Francesco Fanfani, Ramsey D. Badawi, Lorenzo Nardo

**Affiliations:** 1 Nuclear Medicine Unit, G-STeP Radiopharmacy Research Core Facility, Department of Radiology, Radiotherapy and Haematology, Fondazione Policlinico Universitario A. Gemelli IRCCS, 00168 Rome, Italy; 2 Nuclear Medicine Unit, Department of Radiology, Radiotherapy and Haematology, Fondazione Policlinico Universitario A. Gemelli IRCCS, Largo A. Gemelli, 8, 00168 Rome, Italy; 3 Section of Nuclear Medicine, Department of Radiological Sciences, Radiotherapy and Haematology, Università Cattolica del Sacro Cuore, 00168 Rome, Italy; 4 Department of Nuclear Medicine, Inselspital, Bern University Hospital, University of Bern, 3010 Bern, Switzerland; 5 Department of Radiology, University of Pennsylvania, Philadelphia, PA 19104, USA; 6 Woman, Child and Public Health Department, Fondazione Policlinico Universitario A. Gemelli IRCCS, 00168 Rome, Italy; 7 Section of Obstetrics and Gynaecology, University Department of Life Sciences and Public Health, Università Cattolica del Sacro Cuore, 00168 Roma, Italy; 8 Department of Radiology, University of California Davis, Sacramento, CA 95819, USA; 9 Department of Biomedical Engineering, University of California Davis, Davis, CA 95616, USA

**Keywords:** PET/CT, long axial field of view, total body, gynecological, oncology, unmet needs

## Abstract

**Simple Summary:**

Recent advances in PET/CT technology led to the development of long-axial field-of-view (LAFOV) PET/CT with the possibility of simultaneously scanning a large portion of the body with reduced acquisition time, reduced radiation burden, and improved spatial resolution. This article explores the potential applications of LAFOV PET/CT imaging in gynecological malignancies. Patients affected by these diseases are expected to gain multiple and major benefits from this technology and have some of their unmet needs answered in all phases of management, from initial staging to surgery and radiotherapy planning, assessment of treatment response, and restaging, with improved patient-tailored care.

**Abstract:**

Gynecological malignancies currently affect about 3.5 million women all over the world. Imaging of uterine, cervical, vaginal, ovarian, and vulvar cancer still presents several unmet needs when using conventional modalities such as ultrasound, computed tomography (CT), magnetic resonance, and standard positron emission tomography (PET)/CT. Some of the current diagnostic limitations are represented by differential diagnosis between inflammatory and cancerous findings, detection of peritoneal carcinomatosis and metastases <1 cm, detection of cancer-associated vascular complications, effective assessment of post-therapy changes, as well as bone metabolism and osteoporosis assessment. As a result of recent advances in PET/CT instrumentation, new systems now offer a long-axial field-of-view (LAFOV) to image between 106 cm and 194 cm (i.e., total-body PET) of the patient’s body simultaneously and feature higher physical sensitivity and spatial resolution compared to standard PET/CT systems. LAFOV PET could overcome the forementioned limitations of conventional imaging and provide valuable global disease assessment, allowing for improved patient-tailored care. This article provides a comprehensive overview of these and other potential applications of LAFOV PET/CT imaging for patients with gynecological malignancies.

## 1. Introduction

Gynecological malignancies, as defined by the Centers for Disease Control and Prevention (CDC), comprise uterine, cervical, vaginal, vulvar, and ovarian cancers. These neoplasms currently affect about 3.5 million women all over the world [1]. Therefore, they represent a health and social burden that is currently expected to grow as a reflection of both the aging and growth of the population, changes in the prevalence of risk factors, and increased access to screening programs. Incidence and mortality rates depend on geographical variations and changes, along with sociodemographic index values. Data from 2020 surveys in the US [2] are shown in Table 1.

Many medical imaging techniques are currently available for gynecological cancer assessment: ultrasound (US), computed tomography (CT), magnetic resonance imaging (MRI) possibly integrated with diffusion-weighted imaging (DWI), scintigraphy with single-photon emission computed tomography (SPECT) or SPECT/CT, and positron emission tomography (PET)/CT. PET/CT with various radiopharmaceuticals is widely considered the most sensitive imaging technique available for the non-invasive assessment of biologic features and molecular processes of tumors [3,4]. The diagnostic abilities of PET/CT with the glucose analogue fluorine-18 2-fluoro-2-deoxyglucose ([^18^F]FDG) have been widely tested in gynecological oncology for defining disease extent, planning and treatment response, and patient prognosis [5].

In uterine neoplasms, lymph nodes (LNs) are a common site of involvement, mainly localized in the abdominopelvic and retroperitoneal regions [6]. PET/CT has shown 72% sensitivity and 93% specificity for detecting lymph node involvement, better than MRI and CT [7,8,9,10]. In the prospective multicentric study by Gee et al., about 12% of patients with high-risk endometrial cancer demonstrated unsuspected distant metastases at preoperative PET/CT with few false-positive results (98.6% specificity) [11]. 

In locally advanced cervical cancer, pre-treatment PET/CT is currently the standard of care for cancer staging and therapy planning. This procedure is essential for assessing LNs metastases, which may show a normal morphologic pattern at CT or MRI, with a pooled sensitivity and specificity of 82% and 95%, respectively [12], as well as for assessing distant metastases. According to Gee et al., unexpected metastases were detected by [^18^F]FDG PET/CT in 13.7% of patients with locally advanced cervical cancer at initial diagnosis [11].

In vulvar cancer, the major pathway of spread is lymphatic drainage, initially to the inguinofemoral and then to the pelvic LNs, whereas hematogenous distant metastases are rare. According to a recent meta-analysis, which collected studies performed in small and heterogeneous cohorts, per-groin sensitivity and specificity were 76% and 88%, respectively [13]. In a recent large series of vulvar cancer patients, preoperative PET/CT showed good sensitivity (85.6%) and negative predictive value (91.2%) at the groin level in discriminating metastatic from non-metastatic LNs [14]. Compared to MRI and CT, PET/CT is superior in identifying involved pelvic nodes and distant metastases [12]. 

In ovarian cancer, the typical pathway of spread is to the peritoneum and LNs (pelvic, para-aortic, and supraclavicular); distant metastases are less common. According to the International Federation of Gynecology and Obstetrics (FIGO) staging system, staging of ovarian cancer is based on laparotomy or laparoscopy findings [15]. After surgical staging, PET/CT is useful to detect LN metastases and extra-abdominal disease, where it performs better than CT, and to clarify equivocal findings at morphologic imaging [16]. The situation where PET/CT shows high sensitivity and specificity and changes patient management is the detection of recurrent disease, especially in patients with biochemical relapse (elevated Cancer Antigen (CA)-125 levels). In this setting, PET/CT has been reported to perform better than CT and MRI, with pooled sensitivity and specificity of 91% and 88%, respectively, versus 79% sensitivity and 78% specificity for contrast-enhanced (CE)-CT and 75% sensitivity and 78% specificity for MRI [16].

Even though PET/CT plays an important role in the clinical management of patients with gynecological malignancies, several diagnostic needs remain unmet. For instance, differential diagnosis between inflammatory and cancerous findings is still an issue; laparoscopic staging for visual assessment of the entire peritoneal cavity and peritoneal biopsy are often needed for staging and restaging purposes; sentinel lymph node biopsy only partially allows for radical groin lymphadenectomies; and effective assessment of post-therapy changes is often challenging. The responsibility for invasive interventions and other shortcomings falls only to a certain extent on the experience of healthcare centers and is mainly due to imaging limitations and pitfalls [17].

Recent advances in technology have allowed the implementation of long-axial field-of-view (LAFOV) PET/CT systems. Compared to standard-axial field-of-view (SAFOV) systems with a field-of-view (FOV) of 15–30 cm, these new scanners can image 106–194 cm of the patient’s body simultaneously [18] in one bed position (i.e., the anatomical portion of the body being imaged at once in the FOV of the scanner). Moreover, the highly improved technical characteristics of LAFOV PET/CT scanners enable diagnostic imaging to answer clinical unmet needs in many pathological conditions [18], hopefully soon including gynecological malignancies.

In this article, we provide our perspective about the potential role of LAFOV PET/CT in the management of patients with gynecological malignancies, with emphasis on how it could be able to provide answers to some existing unmet diagnostic needs. Such an overview could motivate the currently missing research in gynecological oncology using LAFOV PET/CT. A brief description of the three LAFOV scanners available today is also included in this scientific communication, along with some insight on how their implementation in the clinical routine could represent a breakthrough.

## 2. LAFOV PET/CT Scanners 

Extending the FOV in PET/CT diagnostics was a fundamental step in hybrid imaging after the introduction of digital PET systems [19,20]. 

Different approaches resulted in the development of three LAFOV PET/CT scanners for human imaging, the first one being the uEXPLORER PET/CT [3,21,22]. Within the EXPLORER Consortium (a collaboration between the University of California, Davis, Sacramento, CA, USA, and United Imaging Healthcare, Shanghai, China), the construction of this scanner was completed in the summer of 2018 and used for the first time in human studies in the same year [21]. Its axial FOV is the longest existing for a PET/CT scanner, covering 194 cm. Simultaneous truly total-body (TB) coverage (therefore TB PET) is only provided by this scanner for patients not taller than 194 cm, which is 99% of the world’s population [21,23]. The physics behind the uEXPLORER [24,25] currently makes it the most highly performing commercially available scanner in terms of photon and spatial resolution [25]. 

The EXPLORER Consortium also supported the development of the PennPET Explorer, another LAFOV PET system built and employed since 2020 at the University of Pennsylvania (UPenn, Philadelphia, PA, USA) [26,27] as an academic research project. The first prototype configuration consisted of three ring segments for a total axial FOV length of 64 cm. However, the ring segments are mounted on linear rails to easily allow for the system to be expanded [26], currently reaching an axial FOV of 142 cm plus alignment with a CT [28]. Compared to conventional scanners, the PennPET demonstrated improved image quality (that in PET is influenced by spatial resolution, size of the detector used, noncollinearity of annihilating photons, and range of emitted positrons) and excellent spatial and temporal resolution [27,29,30,31]. 

The most recent of the LAFOV systems is the Siemens Biograph Vision Quadra PET/CT (Siemens Healthineers), first employed at Inselspital, University Hospital of Bern, Switzerland, in October 2020 [32] and now commercially available. In a head-to-head comparison with a conventional scanner, the Siemens Biograph Vision Quadra PET/CT, with its 106 cm axial FOV, demonstrated higher sensitivity (i.e., the number of 511-keV photon pairs per unit time detected by the device for each unit of activity present in a source) and improved image quality, lesion quantification (i.e., applying algorithms to medical images that precisely measure various aspects of an abnormality), and signal-to-noise ratio (i.e., the difference between the lesion and background compared to the noise in the background) [32,33]. 

So far, all three scanners have already shown major implications in the clinic, both for oncological [27,29,30,31,34,35] and non-oncological [27,29,30,31,36,37] applications. 

In Table 2, we provide a summary of the technical characteristics of the three systems that can be found described in detail in other manuscripts [25,26,33,38]. For the scope of the present article, some of the applications and clinical implications that these technical features may specifically have in the field of gynecological oncology will be discussed in the following paragraphs.

**Figure 1:**
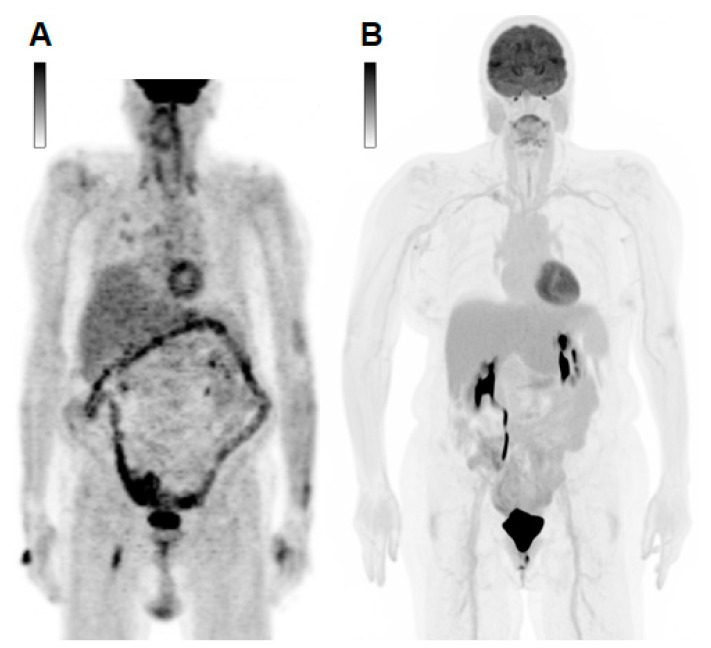
Comparison of imaging from different generations of PET/CT scanners. Maximum intensity projections (MIP) of a conventional analogue scanner with a 60 min acquisition time in 2002 (**A**) and a state-of-the-art long-axial field-of-view (LAFOV) digital scanner with a 6 min acquisition time in 2021 (**B**).

An example image shows the difference in spatial resolution, sensitivity, and acquisition time between a conventional analogue scanner in 2002 and a state-of-the-art 2021 LAFOV system (Figure 1).

## 3. Diagnostic Unmet Needs in Gynecological Malignancies

### 3.1. Assessment of Disease Extent and Quantitative Global Disease Assessment

After the clinical (patient’s history, physical examination, and lab tests) and histological diagnosis of cancer, defining the extent of the disease is a primary concern in oncology. Different imaging approaches should be considered and selected for specific issues. 

Generally, gynecologists perform pelvic transvaginal US as a first-level examination in combination with pelvic palpation. For uterine cancer, pelvic MRI, which provides excellent soft tissue contrast, can be considered the gold standard for defining the local extension of the tumor, which is essential for surgical planning [39]. Despite the high accuracy of US, MRI is frequently used for adnexal mass evaluation with the aim of better defining the risk of malignancy and the anatomical relationship with pelvic organs. However, as far as LNs assessment is concerned, MRI has low sensitivity (about 50%) [10]. This issue is relevant since the presence of LNs metastases could drastically change the treatment approach between upfront surgery or chemo-radiation in cervical cancer or can require abdominal vs. minimally invasive surgery for endometrial and ovarian cancer patients, in which lymphadenectomy moves from staging to a cytoreductive step.

MRI is often followed by a CE-CT scan in cases of high-grade uterine carcinoma, in cervical carcinoma with revised 2018 FIGO [40] stages IB1–IB3, in vaginal and ovarian cancer when there is a clinical indication, and in vulvar cancer for T2 or larger primary tumor or when metastases are suspected. Guidelines require at least the abdomen and pelvis to be examined through CE-CT, while an X-ray is considered enough for thorax examination, unless differently indicated by the clinician and/or if distant metastases are suspected. In these cases, CE-CT is often supported by [^18^F]FDG PET/CT, which may be considered an alternative to CE-CT in particular clinical situations (essentially when CE-CT is contraindicated) and when metastatic disease is suspected.

The main indications for [^18^F]FDG PET/CT in gynecological tumors, according to current guidelines and literature evidence [12,39,41,42,43,44], are listed in Table 3.

In light of the great imaging potential that LAFOV PET/CT offers, regional assessment of cancer has limited value in the overall diagnosis and management of such serious and systemic human illnesses [45]. Indeed, simultaneous imaging of larger portions of the body, between 106 and 194 cm, opens the possibility of global disease assessment. In this regard, LAFOV PET/CT imaging is likely to be superior to all other conventional modalities, which individually do not reflect a measurement of overall disease activity. LAFOV scanners also allow dynamic imaging, providing simultaneous time-activity curves for organs or lesions not contained within the conventional PET/CT FOV. Entire body kinetics can be reconstructed and analyzed to create parametric images that may offer complementary information to typical Standardized Uptake Value (SUV)-scaled images [34]. Furthermore, LAFOV imaging has enhanced the potential role of global disease quantification and may be the right modality to enable quantification of a patient’s global disease burden [45]. Such data will represent an accurate simultaneous calculation of regional activity at all disease sites, a reduction in underestimation of regional and global values related to respiratory and cardiac motions, and the interaction between various biological systems. Finally, global disease quantification may be used to test and validate the efficacy of novel (pharmaceutical) interventions [45]. 

LAFOV scanners, thanks to their improved spatial resolution and sensitivity and their ability to offer dynamic imaging, may in the future become an alternative to surgical staging, providing detailed information regarding all relevant organs and lesions simultaneously in an extended field of view [46].

### 3.2. Differential Diagnosis between Physiological, Inflammatory, Benign, and Malignant Findings

In PET/CT studies with [^18^F]FDG, this radiolabeled glucose analogue accumulates in all metabolically active cells, both benign (e.g., the highly glucose-avid brain cells) and malignant (i.e., dividing cancer cells) in nature. Inflammatory and other physiologic biological processes also require glucose consumption, which can be detected by [^18^F]FDG PET/CT. 

Many normal metabolic and structural changes occur in the female reproductive tract in pre- and post-menopausal women throughout their life span and periodically during the menstrual cycle. [^18^F]FDG PET/CT can detect these physiologic variations, especially in the uterus and ovaries. Such patterns, which are well described in the article by Dejanovic et al. [17], may be responsible for possible false-positive findings; therefore, being aware of them may avoid misinterpretation of PET images. Some examples include functional (on a hormonal basis) ovarian [^18^F]FDG uptake during the late follicular and early luteal phases of the menstrual cycle and functional uptake in the endometrial cavity during the ovulatory and menstrual phases [47]. Another possible false-positive finding is the accumulation of urine activity in the ureters, mimicking lymph node metastases. Moreover, physiological bowel or bladder activity can mask peritoneal lesions, causing false negative results [17]. In addition to physiologic uptake, many other processes may demonstrate increased glucose metabolism. For example, uterine leiomyomas, which are the most common benign uterine neoplasms, show highly variable FDG uptake and can be difficult to differentiate from uterine carcinomas or sarcomas. Inflammatory and infectious processes too, or other benign conditions such as endometriotic implants and ureteral diverticula, may be detected as areas of increased [^18^F]FDG uptake and be misdiagnosed as tumors. Several other pitfalls have been described [48]. However, the most common interpretation issue in this setting is probably represented by the differentiation between malignant and benign LNs. The use of delayed and dynamic (thus also parametric) imaging allowed by LAFOV PET/CT scanners may help the nuclear medicine physician avoid misdiagnosing. Indeed, based on experience gained by several research groups, it has become clear that FDG uptake in tumor lesions increases over time and reaches a plateau at 4 to 5 h, and several types of tumors have an uptake peak significantly later than the conventional 1-h uptake time used on SAFOV scanners [45,49,50]. At these delayed time points, there is a significant physiologic clearance of the radiotracer from organs and tissues such as the liver, kidneys, bladder, and blood, leading to an increase in tumor-to-background ratios. The improved sensitivity of LAFOV systems allows for delayed imaging with images of diagnostic quality and relatively low background noise, which may improve lesion detection and enable fundamental biologic insights, as described by Pantel et al. [27]. By testing the uptake curve of certain tissues and sites in “dual phase” acquisition protocols, a major impact could be observed in the optimal management of oncological patients, especially when the exact location and extent of the tumor are fundamental for surgical interventions or radiation therapy planning [12]. Some attempts at dual-time imaging have been made with conventional scanners, but with various limitations and few applications [51,52]. It could also be hypothesized that, in some cases, a delayed imaging timepoint will show previously unseen metastatic sites with different kinetics, suggesting biologic heterogeneity of the tumor, as previously described in other tumor entities like prostate cancer [27,53]. LAFOV PET/CT imaging may provide a full overview of tumor biological heterogeneity, guiding the performance of targeted biopsies or allowing for a reduction in their number, which are frequently and invasively performed in gynecological cancers. In this regard, LAFOV scanners have also shown potential for imaging more than one molecular target at the same time with dual-tracer examination protocols [54]. This kind of examination is still an unmet need in the evaluation and introduction of new radiopharmaceuticals in clinical practice. These novel radiopharmaceuticals, which explore specific biological characteristics of tumors, are currently under evaluation in gynecological cancers with the potentiality of identifying new prognostic factors and/or new personalized therapies. Among these, fibroblast activation protein inhibitors (FAPI) PET/CT [55] and fluoro-estradiol (FES) PET/CT [10,56] have shown preliminary promising results in gynecological cancer.

**Figure 2:**
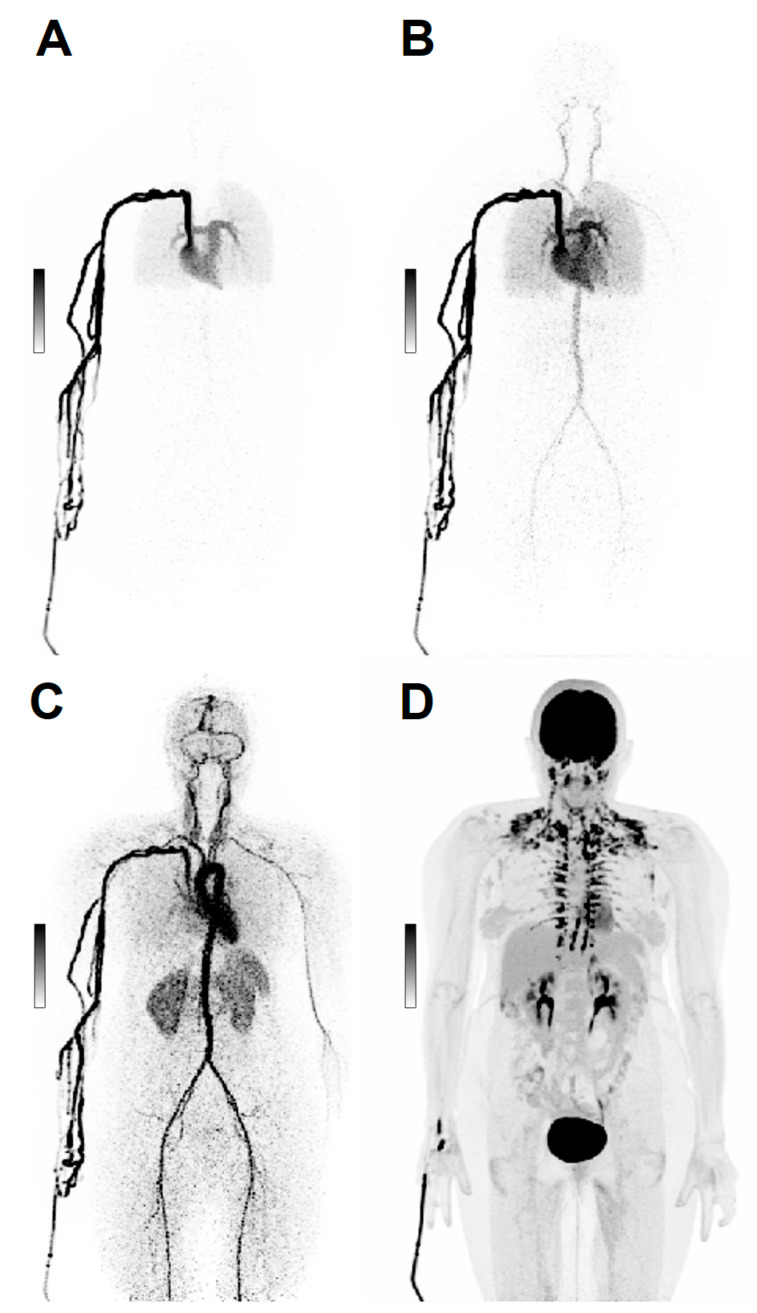
Dynamic Imaging. Maximum intensity projections (MIP) of a 69 y/o patient with ovarian cancer, scanned dynamically with 168 MBq [^18^F]FDG. After injection, the first 2 min of acquisition show the early biodistribution of the radiopharmaceutical (**A**–**C**). Image (**D**) shows [^18^F]FDG distribution at 60 min post-injection.

LAFOV technology has also opened new perspectives in dynamic imaging [34,57], providing simultaneous time-activity curves for organs and lesions that would not be included altogether in the FOV of conventional scanners [34], avoiding the need for simultaneous blood sampling (Figure 2).

On a simple level, the dynamic acquisition of PET images makes it possible to clearly identify the [^18^F]FDG elimination path and thus distinguish with greater certainty the urine uptake in the bladder and ureters from neighboring sites of non-physiological uptake [58]. In the future, dynamic imaging may even become a complementary solution to conventional imaging protocols offered by LAFOV scanners. Indeed, the dynamic acquisition kinetic information may prove valuable for precision medicine. Entire body kinetics datasets can be reconstructed and analyzed to create parametric images that may offer complementary information to typical SUV-scaled images [34] and improve quantification [59]. Utilization rate (Ki) images of [^18^F]FDG can be provided to visualize glucose utilization in the target lesion, not confounded by contributions from [^18^F]FDG in plasma [59]. This kinetic information can be applied to identify small regions of tumoral infiltration, as such lesions may lack adequate contrast for detection in SUV images but sufficiently alter the kinetics within a voxel that they can be appreciated in parametric images [38]. Such a paradigm allows for the functional characterization of low-grade disease (cancers, inflammation/infection) that has so far represented a pitfall in [^18^F]FDG imaging [18]. Abbreviations of LAFOV scan protocols might allow overcoming the challenge of current long scan duration in dynamic imaging and might possibly lead to the implementation of dynamic imaging models into the clinical routine [34,60]. 

### 3.3. Detection of Peritoneal Carcinomatosis and Sub-Centimetric Metastases

Early detection of peritoneal carcinomatosis (i.e., seeding of metastatic implants in the peritoneal cavity) is an important step in staging and restaging gynecological malignancies, mainly ovarian cancer, as it is essential for complete cytoreductive surgery and prognostic information [61]. As already mentioned, the presence of peritoneal carcinomatosis in gynecological cancers is often assessed through surgical inspection. Anatomic imaging with CE-CT and MRI (also using DW sequences) plays an important role in non-invasively assessing peritoneal metastases. However, detection of peritoneal carcinomatosis is highly influenced by lesion size and site and the presence of ascites [61]. According to a recent meta-analysis, MRI and standard [^18^F]FDG PET/CT show similar diagnostic performance for the detection of peritoneal metastases in patients with ovarian cancer, with a pooled sensitivity and specificity of 92% and 85%, respectively, for DW-MRI and 80% and 90%, respectively, for PET/CT, with sensitivity values exceeding those of CT (68% sensitivity and 88% specificity) [62]. At PET/CT, patterns of visualization of peritoneal carcinomatosis include a focal pattern, with single or multiple areas of hypermetabolic activity that may become confluent, such as those involving paracolic gutters, small bowel mesentery, the pouch of Douglas, or the abdominal cavity among bowel loops; or a diffuse pattern with diffuse thickening and intense uptake, usually seen along the liver capsule or in the greater omentum (omental cake) [63]. These and other sites of metastases may go undetected on conventional PET/CT scanners if they are smaller than 1 cm in size, which is a major flaw associated with SAFOV [^18^F]FDG imaging.

**Figure 3:**
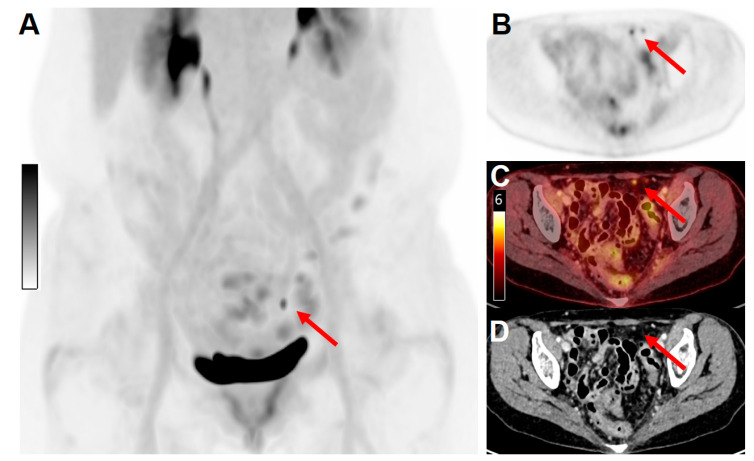
Example of the high sensitivity and spatial resolution of a long-axial field-of-view scanner. A 64 y/o female patient with cervical cancer was treated with surgery (hysterectomy and pelvic lymphadenectomy), followed by adjuvant radiation therapy, and systemic treatment with cisplatin. For response-to-treatment assessment, the patient underwent a PET/CT examination on the Biograph Vision Quadra scanner. (**A**) Maximum intensity projection (MIP) of images acquired 60 min after injection of 130 MBq [^18^F]FDG. (**B**) PET only, (**C**) fused PET/CT, and contrast-enhanced CT (**D**) images revealed the presence of two peritoneal metastatic lesions (red arrows, maximum diameter 4 mm) with clearly distinguishable [^18^F]FDG uptake (SUV_max_ 4.6).

The ultra-high sensitivity and the possibility to perform delayed imaging of LAFOV scanners have demonstrated higher diagnostic accuracy outputs compared to conventional PET imaging [27,34,35,45,46], allowing for the detection of small tumor deposits. Higher count statistics resulting in an improvement of signal-to-noise and tumor-to-background ratios simplify the detection of obscure small lesions [33,64] (Figure 3). 

If a downside may be pointed out, normal structures could also appear in a redefined way on LAFOV PET/CT images [18,34]. Hopefully, the implementation of LAFOV scanners in clinical settings will increase the nuclear medicine physicians’ experience in reading such high-definition images and avoid the risk of an increased number of false-positive results.

### 3.4. Effective Assessment of Post-Therapy Changes

Imaging techniques such as CT and MRI (even when integrated with DWI) cannot reliably distinguish residual disease from post-therapy changes (e.g., post-surgery and post-radiation therapy). A major flaw associated with this limitation is the generation of false-positive cases. In this setting, [^18^F]PET/CT has an excellent negative predictive value, while the number of false-positive results is still not optimal. In general, a 3-month interval is recommended for a reliable post-therapy functional assessment, as at this time radio-induced inflammation is thought to be resolved. Minimal residual disease and post-therapeutic local changes may have very different kinetic parameters that could be potentially detected by using parametric maps created by LAFOV PET datasets. This approach may in the future help to differentiate inflammation from tumor [18]. 

### 3.5. Motion Artifacts

Imaging on SAFOV PET/CT scanners requires between 15 and 20 min for a whole-body acquisition (skull-base to mid-thighs). During this time, the patient’s movements can lead to motion artifacts and disalignment between the PET and the CT images, with a consequent increased risk of misreadings. Other involuntary movements that may cause artifacts and interfere with image reading are represented by respiratory motion and heartbeat, bowel motion, and bladder filling with radioactive urine. Such issues are not to be underestimated in patients with a gynecological malignancy, as the pelvis represents a complex anatomical district with sites of physiological metabolic activity that may overlap with tumor lesions and vice versa, leading to false-positive or false negative results. LAFOV PET/CT scanners allow the implementation of fast and even ultra-fast acquisition protocols; the Biograph Vision Quadra system provided diagnostic-quality images, comparable to those of a 16-min scan on a SAFOV PET/CT, in 2 min [32]. For European Association of Nuclear Medicine Research Ltd. (EARL) standard compliant acquisition and reconstruction protocols, scan durations on the Biograph Vision Quadra could even be reduced to 1 min [64]. The uEXPLORER scanner provides high-quality images in an acquisition time of even 0.5 min, as found in the results of the NCT04110743 clinical trial (that has low-dose PET/CT scans, TB perfusion, and early biodistribution understanding in healthy volunteers among its aims) and further stated in an expert consensus on [^18^F]FDG PET/CT TB imaging [23]. Moreover, decreased acquisition time leads to a reduction in the need for sedation in debilitated patients, claustrophobic patients, and patients with difficulty laying still due to neurological, respiratory, or pain reasons, which would otherwise most commonly represent the cases in which motion artifacts interfere with correct imaging [46].

### 3.6. Radiation Exposure

One of the clear advantages of LAFOV PET/CT imaging and one of its most appreciated features is the already mentioned possibility to reduce the administered radiopharmaceutical activity down to a tenth of what is needed on conventional scanners and still obtain high-quality diagnostic images. The follow-up of oncological patients requires the repetition of the same examinations performed at baseline, for the correct interpretation of anatomical (and functional) changes, response to therapies, or evaluation of disease relapse. With LAFOV PET/CT imaging, patients will be able to undergo multiple scans for disease re-assessment with a substantial decrease in administered activity when compared to using conventional PET/CT systems [46,65]. An example can be found in the natural history of cervical cancer, which, in approximately one-third of patients, recurs within the first 2 years after therapy. The sites of recurrence are the vaginal vault (better distinguishable through dynamic acquisition), parametrial and pelvic walls, para-aortic and supraclavicular lymph nodes, and distant metastases, including peritoneal disease. The current survival rate is low, and cure efficacy is minimal in these patients [66]. Low-dose and more frequent follow-up PET/CT exams on LAFOV scanners could increase the early detection of recurrence and improve patients’ survival.

Ultra-low-dose protocols are already in use [23], and it can be expected that many applications will follow. In some cases, even their use for screening purposes has been hypothesized [3,67]. Subjects with a genetic-familial high-risk for ovarian cancer may be among the possible recipients of such an application, as the appropriateness of imaging modalities and scheduling in these patients is still under study [68].

Another of the most appealing current areas of interest for ultra-low-dose PET/CT exams is pregnancy. Cancer is diagnosed in approximately one in every 1000 pregnancies annually, with melanoma, breast cancer, cervical cancer, lymphomas, and leukemia being the most commonly associated malignancies [69]. LAFOV PET/CT could allow these patients to have a TB functional oncological assessment—and the best possible disease management—at unprecedented low-risk exposure to radiation.

Moreover, it is now being hypothesized that diagnostic PET images alone can be obtained with no or very low-dose CT (currently needed for attenuation correction) through deep learning and new reconstruction protocols [3,67,70,71,72].

### 3.7. Pain

One of the issues most associated with cancer is pain. Gynecological oncological patients can experience it too, especially in cases of bone metastases. The incidence of bone metastases in endometrial cancer is reported to be 6 to 15% [73,74], approximately 1.2% in ovarian cancer [75], and 1.1% in cervical neoplasms [76]. Improvements in surgery, radiation, and the development of novel chemotherapeutic agents have led to prolonged survival and an increase in the prevalence of bone metastases. Pain may prevent patients from undergoing important examinations like PET/CT. In these cases, even if the assessment leads to palliative treatments, it is still of utmost importance for physicians to put their effort into offering patients a solution for their dignity and quality of life. LAFOV PET/CT can represent a valuable solution because it can significantly shorten the acquisition time while maintaining good detectability of lesions and diagnostic image quality [77].

### 3.8. Detection of Cancer-Associated Vascular Complications

Oncological patients experience cancer-associated vascular complications, with a high incidence reported for both deep vein thrombosis and pulmonary embolism, up to a seven-fold increased risk compared to the non-oncological population [78]. Gynecological oncology patients are also affected and can experience recurrence of venous thromboembolism and/or pulmonary embolism despite treatment with edoxaban or a vitamin K antagonist, with no significant difference in outcomes among the different tumor types [79]. Cancer-associated vascular complications may result in an interruption of treatments or premature death. As conventional PET/CT fails to image the lower extremities, common sites of venous thrombosis often go unassessed, and clots go undetected. A delayed imaging of the total body may detect increased [^18^F]FDG uptake in blood clots as blood pool activity decreases in the venous system. Thus, delayed TB PET/CT imaging could act as a means of detection and may have a huge impact on the management of cancer patients and their outcome by identifying the clots earlier and with greater sensitivity [18,45].

### 3.9. Bone Metabolism and Osteoporosis Assessment

Women with gynecological cancer have an increased risk of cancer treatment-induced bone loss, which impacts their quality of life and overall survival [80]. Unfortunately, current imaging techniques have several limitations in the reliable assessment of bone metabolism and fail to detect a decreased bone mineral density at early time points. The currently most adopted modality is dual-energy x-ray absorptiometry (DXA), which, however, implies overestimating the results. Sodium fluoride (NaF) PET/CT is a promising imaging tool to fill this need [45], and its association with the already thoroughly discussed TB imaging advantages could represent the source of major benefits for patients.

## 4. Discussion

The most identifying feature of LAFOV scanners is their capability of imaging multiple organs and systems simultaneously during the same phases of radiotracer distribution and uptake, with an increase in signal collection efficiency at slightly variable magnitudes across the three systems described. The sensitivity gain (15–68 fold higher than that of a conventional scanner [25]), essential for detecting and quantifying even small pathological processes, can allow the acquisition of diagnostic-quality images with very small amounts of radioactivity in the FOV (down to 0.37 MBq/kg vs. 3.7 MBq/kg typically needed in a SAVOF PET/CT), with very short acquisition times (down to 0.5 min vs. 15–20 min typically needed on a conventional scanner), at later time points after the radiotracer’s administration, or a combination of these [21,23]. 

The possibility of scanning patients on LAFOV systems with low activity was confirmed in several studies. For example, the initial results of an ongoing clinical study (ClinicalTrials.gov identifier NCT04110743) demonstrated the feasibility of scanning adult subjects with doses as low as 0.5 mCi at 90 min post-injection. The decrease in injected dose drastically reduces the exposure of the patients, maintaining images of diagnostic quality to fulfill the As-Low-As-Reasonably-Achievable (ALARA) principle. This achievement is particularly useful in the pediatric population and in oncological subjects that will have several longitudinal follow-up studies in their lifetime. 

The possibility of delayed imaging has been demonstrated to be critical for the detection of both physiologic and pathologic processes. For example, it allows for the clearance of radiotracer from several organs and background tissues (e.g., liver, blood pool, muscle), helping the visualization of tumor lesions, which usually present persistent uptake even several hours post-radiotracer administration. Using the PennPET LAFOV scanner, Pantel et al. demonstrated better visualization of peritoneal lesions when images acquired 2.75 and 4.2 h post-injection were compared to images acquired at the standard 60 min post-injection [27]. The increase in signal-to-background ratio for hard-to-detect lesions such as peritoneal lesions is related to the washout of surrounding anatomical structures and the kinetics of the described metastatic lesions. A similar concept can be applied to the pelvic region, where the excretion of radiotracer in the bladder often creates artifacts and limits the visualization of surrounding findings. In this setting, delayed scanning allows for clearance and/or dilution of radiotracer from/in the bladder, which in turn allows for decreasing artifacts and better lesion assessment in this area. Moreover, a LAFOV PET/CT study recently showed that there is physiologic gallbladder [^18^F]FDG uptake in most of the healthy and oncologic subjects scanned at 2 h or later, which should not be confused for inflammation (cholecystitis) or tumor, especially in patients diagnosed with a malignancy that is likely to spread in the peritoneum [81]. Delayed scanning is limited in conventional PET scanners, mainly because of their low sensitivity, which results in significant background noise that degrades image quality. 

The high sensitivity of LAFOV scanners allows for a decrease in the acquisition time of LAFOV PET/CT imaging protocols. For example, at the University of Bern, scans are acquired with the Biograph Vision Quadra PET/CT using a 4–6-min acquisition time. This decreases patients’ discomfort of lying on the scanner, and it is even more valuable in patients in pain or young patients that cannot hold still for the typical 15–25 min PET/CT acquisition time for eyes-to-thighs (i.e., whole-body) protocols.

Many ways exist to exploit the increase in signal collection efficiency, overcoming the limitations of conventional PET scanners’ hardware and technology [82]. Overall, LAFOV PET allows patient-tailored imaging protocols [23]. However, the installation and implementation of LAFOV PET/CT have high costs that make them unaffordable for many hospitals, even in high-income countries [67]. If, on the one hand, a realistic business plan could focus on the possibility of increasing patient throughput, on the other hand, there would be multiple parallel aspects to be considered. Infrastructure, radiochemistry, patient logistics, facilities, additional personnel, extension of the catchment area, and PET usage implementation in other populations (e.g., healthy volunteers) or using tracers other than [^18^F]FDG, are some of these aspects. LAFOV scanners would also be a great incentive for the integration of research protocols into the clinical routine. All motion artifacts and kinetic modeling challenges would need to be tackled. Storing and processing enormous LAFOV PET/CT datasets would be a challenge for the information technology infrastructure. Nevertheless, it is expected that these practical issues will be resolved soon [67].

In gynecological malignancies, some social considerations might be worth a thought. Future implementation of LAFOV scanners in high-income countries will inevitably lead to an increase in the inequality of medical supply in already underprivileged countries. Indeed, survival disparities between women from different geographical regions are influenced by various factors across the spectrum of care. Those include timely access to guideline-concordant care and clinical trial enrollment [83]. For instance, endometrial cancer has rising mortality rates, driven by the increasing incidence of high-risk histologic subtypes, disproportionately affecting black women [83]. The lack of progress made in endometrial cancer treatment, particularly for high-risk histologic subtypes, could become a cue for LAFOV PET/CT users to include these populations in clinical studies for the benefit of science and society.

## 5. Conclusions

LAFOV PET/CT imaging may have a revolutionary impact on the day-to-day practice of medicine and may become a leading imaging modality in the future. Indeed, its main accomplishments are represented by:

- larger FOV (up to total-body coverage);
- higher scanner sensitivity and consequently improved image quality, demonstrating sites of uptake previously unseen on conventional PET that may result in earlier detection of tumor lesions;
- possibility of lower radiotracer activity administration, with decreased overall radiation exposure of patients, or shorter image protocol acquisition, with increased tolerability of imaging;
- dynamic scanning, with the attainment of non-invasive kinetic modeling and better quantification;
- delayed scanning, which may improve the visualization of tumors by increasing the ratio between tumor uptake and background.

Patients affected by gynecological malignancies can have multiple and major benefits from this technology and finally have some of their unmet needs answered.

The flexibility of LAFOV PET/CT imaging protocols allows for much improved patient-tailored care.

## Figures and Tables

**Table 1:** Gynecological cancers: incidence and mortality rates *.

Cancer Type	Incidence Rate (/100.000 Women/year)	Death Rate (/100.000 Women/year)	Diagnoses at Local Disease Stage (%)	Diagnoses at Loco-Regional Spread Stage (%)	Diagnoses at Metastatic Stage (%)	Mean 5-Year Relative Survival Rate (%)
**Uterine**	27.8	5.1	67.0	20.0	9.0	81.3
**Cervical**	7.8	2.2	44.0	36.0	16.0	66.7
**Vaginal**	2.0	Na	66.0	54.0	24.0	49.0
**Vulvar**	2.6	0.6	60.0	28.0	6.0	70.3
**Ovarian**	10.6	6.3	17.0	21.0	57.0	49.7

* [SEER Cancer Stat Facts. National Cancer Institute, Bethesda. https://seer.cancer.gov/statfacts/ (accessed on 31 January 2023)]. Na: data not available.

**Table 2:** Technical characteristics of currently available LAFOV PET/CT scanners.

Scanner	uEXPLORER	PennPET Explorer	Biograph Vision Quadra
**Company**	UC Davis, United Imaging Healthcare	University of Pennsylvania	Siemens Healthineers
**Bore diameter (cm)**	76	76.4	78
**Scintillator** - Size (mm)	LYSO 2.76 × 2.76 × 18.1	LYSO 3.86 × 3.86 × 19	LSO 3.2 × 3.2 × 20
**Photo-sensors**	Analogue SiPMs	Digital SiPMs	Digital SiPMs
**Axial field-of-view (cm)**	194	142	106
**Time of flight resolution (ps)**	505	250	228
**Sensitivity (kcps/MBq)**	174	140	176
**Spatial resolution (mm)**			
- Axial	2.9	4.0	3.4
- Transaxial	3.0	4.0	3.4
**CT slice**	160	128	128

LYSO: Cerium-doped Lutetium Yttrium Orthosilicate crystal; SiPMs: silicon photomultiplier; Time of flight: the difference in the arrival times of the two photons on paired detectors; Sensitivity: the number of 511-keV photon pairs per unit time detected by the device for each unit of activity present in a source; CT: computed tomography.

**Table 3:** Main indications for [^18^F]FDG PET/CT in gynecological tumors.

Cancer Type	Indications
Uterine	(a) Endometrial carcinoma: initial workup, when metastatic disease is suspected in selected patients; suspected recurrence; for postoperative radiotherapy target delineation in patients at high risk of recurrence, especially if no nodes were sampled; for radiotherapy treatment planning in medically inoperable patients planned for definitive radiation by brachytherapy alone. (b) Uterine sarcoma: initial workup, to clarify ambiguous findings; follow-up/post-treatment surveillance, if metastatic disease is suspected.
Cervical	Initial workup of patients with revised 2018 FIGO stage >IB1, to evaluate nodal and distant disease; radiation therapy planning; response to treatment assessment in women with FIGO stage IB3 to IVA disease; recurrence; surveillance.
Vaginal	Initial workup, when metastatic disease is suspected in selected patients; follow-up/post-treatment surveillance; suspected recurrence.
Vulvar	Initial workup of patients with ≥T2 tumors or if metastatic disease is suspected; follow-up/surveillance if recurrence/metastasis is suspected; follow-up/surveillance, to assess treatment response if primary treatment was with definitive intent; follow-up/surveillance, in cases of high-risk disease every 4–12 months.
Ovarian	Initial workup in newly diagnosed ovarian cancer after a recent surgical procedure, if clinically indicated; stages II–IV post-primary treatment; follow-up/post-treatment surveillance in patients with rising Cancer Antigen-125 levels and equivocal findings at anatomic imaging studies for detection of recurrent disease; therapy monitoring.

## Data Availability

Not applicable.

## References

[1] Sung H., Ferlay J., Siegel R.L., Laversanne M., Soerjomataram I., Jemal A., Bray F. (2021). Global Cancer Statistics 2020: GLOBOCAN Estimates of Incidence and Mortality Worldwide for 36 Cancers in 185 Countries. CA Cancer J. Clin..

[2] National Cancer Institute, Bethesda SEER Cancer Stat Facts. https://seer.cancer.gov/statfacts/.

[3] Cherry S.R., Jones T., Karp J.S., Qi J., Moses W.W., Badawi R.D. (2018). Total-Body PET: Maximizing Sensitivity to Create New Opportunities for Clinical Research and Patient Care. J. Nucl. Med..

[4] Townsend D.W. (2008). Combined Positron Emission Tomography–Computed Tomography: The Historical Perspective. Semin. Ultrasound CT MRI.

[5] Salem A.E., Fine G.C., Covington M.F., Koppula B.R., Wiggins R.H., Hoffman J.M., Morton K.A. (2022). PET-CT in Clinical Adult Oncology-IV. Gynecologic and Genitourinary Malignancies. Cancers.

[6] Kilcoyne A., Chow D.Z., Lee S.I. (2019). FDG-PET for Assessment of Endometrial and Vulvar Cancer. Semin. Nucl. Med..

[7] Kakhki V.R.D., Shahriari S., Treglia G., Hasanzadeh M., Zakavi S.R., Yousefi Z., Kadkhodayan S., Sadeghi R. (2013). Diagnostic Performance of Fluorine 18 Fluorodeoxyglucose Positron Emission Tomography Imaging for Detection of Primary Lesion and Staging of Endometrial Cancer Patients: Systematic Review and Meta-Analysis of the Literature. Int. J. Gynecol. Cancer.

[8] Lee H.J., Park J.-Y., Lee J.J., Kim M.H., Kim D.-Y., Suh D.-S., Kim J.-H., Kim Y.-M., Kim Y.-T., Nam J.-H. (2016). Comparison of MRI and 18F-FDG PET/CT in the Preoperative Evaluation of Uterine Carcinosarcoma. Gynecol. Oncol..

[9] Shweel M.A., Abdel-Gawad E.A., Abdel-Gawad E.A., Abdelghany H.S., Abdel-Rahman A.M., Ibrahim E.M. (2012). Uterine Cervical Malignancy: Diagnostic Accuracy of MRI with Histopathologic Correlation. J. Clin. Imaging Sci..

[10] Paredes P., Paño B., Díaz B., Vidal-Sicart S., Collarino A., Vidal-Sicart S., Olmos R.A.V. (2022). Endometrial Cancer. Nuclear Medicine Manual on Gynaecological Cancers and Other Female Malignancies.

[11] Gee M.S., Atri M., Bandos A.I., Mannel R.S., Gold M.A., Lee S.I. (2018). Identification of Distant Metastatic Disease in Uterine Cervical and Endometrial Cancers with FDG PET/CT: Analysis from the ACRIN 6671/GOG 0233 Multicenter Trial. Radiology.

[12] Rao Y.J., Grigsby P.W. (2018). The Role of PET Imaging in Gynecologic Radiation Oncology. PET Clin..

[13] Triumbari E.K.A., de Koster E.J., Rufini V., Fragomeni S.M., Garganese G., Collarino A. (2021). 18F-FDG PET and 18F-FDG PET/CT in Vulvar Cancer: A Systematic Review and Meta-Analysis. Clin. Nucl. Med..

[14] Rufini V., Garganese G., Ieria F.P., Pasciuto T., Fragomeni S.M., Gui B., Florit A., Inzani F., Zannoni G.F., Scambia G. (2021). Diagnostic Performance of Preoperative [18F]FDG-PET/CT for Lymph Node Staging in Vulvar Cancer: A Large Single-Centre Study. Eur. J. Nucl. Med. Mol. Imaging.

[15] Prat J. (2015). FIGO Committee on Gynecologic Oncology Staging Classification for Cancer of the Ovary, Fallopian Tube, and Peritoneum: Abridged Republication of Guidelines From the International Federation of Gynecology and Obstetrics (FIGO). Obstet. Gynecol..

[16] Kemppainen J., Hynninen J., Virtanen J., Seppänen M. (2019). PET/CT for Evaluation of Ovarian Cancer. Semin. Nucl. Med..

[17] Dejanovic D., Hansen N.L., Loft A. (2021). PET/CT Variants and Pitfalls in Gynecological Cancers. Semin. Nucl. Med..

[18] Katal S., Eibschutz L.S., Saboury B., Gholamrezanezhad A., Alavi A. (2022). Advantages and Applications of Total-Body PET Scanning. Diagnostics.

[19] Alberts I., Prenosil G., Sachpekidis C., Weitzel T., Shi K., Rominger A., Afshar-Oromieh A. (2020). Digital versus Analogue PET in [68 Ga] Ga-PSMA-11 PET/CT for Recurrent Prostate Cancer: A Matched-Pair Comparison. Eur. J. Nucl. Med. Mol. Imaging.

[20] Alberts I., Sachpekidis C., Prenosil G., Viscione M., Bohn K.P., Mingels C., Shi K., Ashar-Oromieh A., Rominger A. (2021). Digital PET/CT Allows for Shorter Acquisition Protocols or Reduced Radiopharmaceutical Dose in [18F]-FDG PET/CT. Ann. Nucl. Med..

[21] Badawi R.D., Shi H., Hu P., Chen S., Xu T., Price P.M., Ding Y., Spencer B.A., Nardo L., Liu W. (2019). First Human Imaging Studies with the EXPLORER Total-Body PET Scanner. J. Nucl. Med. Off. Publ. Soc. Nucl. Med..

[22] Cherry S.R., Badawi R.D., Karp J.S., Moses W.W., Price P., Jones T. (2017). Total-Body Imaging: Transforming the Role of Positron Emission Tomography. Sci. Transl. Med..

[23] Yu H., Gu Y., Fan W., Gao Y., Wang M., Zhu X., Wu Z., Liu J., Li B., Wu H. (2022). Expert Consensus on Oncological [18F]FDG Total-Body PET/CT Imaging (Version 1). Eur. Radiol..

[24] Lv Y., Lv X., Liu W., Judenhofer M.S., Zwingenberger A., Wisner E., Berg E., McKenney S., Leung E., Spencer B.A. (2019). Mini EXPLORER II: A Prototype High-Sensitivity PET/CT Scanner for Companion Animal Whole Body and Human Brain Scanning. Phys. Med. Biol..

[25] Spencer B.A., Berg E., Schmall J.P., Omidvari N., Leung E.K., Abdelhafez Y.G., Tang S., Deng Z., Dong Y., Lv Y. (2021). Performance Evaluation of the UEXPLORER Total-Body PET/CT Scanner Based on NEMA NU 2-2018 with Additional Tests to Characterize PET Scanners with a Long Axial Field of View. J. Nucl. Med. Off. Publ. Soc. Nucl. Med..

[26] Karp J.S., Viswanath V., Geagan M.J., Muehllehner G., Pantel A.R., Parma M.J., Perkins A.E., Schmall J.P., Werner M.E., Daube-Witherspoon M.E. (2020). PennPET Explorer: Design and Preliminary Performance of a Whole-Body Imager. J. Nucl. Med..

[27] Pantel A.R., Viswanath V., Daube-Witherspoon M.E., Dubroff J.G., Muehllehner G., Parma M.J., Pryma D.A., Schubert E.K., Mankoff D.A., Karp J.S. (2020). PennPET Explorer: Human Imaging on a Whole-Body Imager. J. Nucl. Med..

[28] https://www.med.upenn.edu/pennpetexplorer/scanner-progress.html.

[29] Rodriguez J.A., Selvaraj S., Bravo P.E. (2021). Potential Cardiovascular Applications of Total-Body PET Imaging. PET Clin..

[30] Chaudhari A.J., Raynor W.Y., Gholamrezanezhad A., Werner T.J., Rajapakse C.S., Alavi A. (2021). Total-Body PET Imaging of Musculoskeletal Disorders. PET Clin..

[31] Saboury B., Morris M.A., Nikpanah M., Werner T.J., Jones E.C., Alavi A. (2020). Reinventing Molecular Imaging with Total-Body PET, Part II: Clinical Applications. PET Clin..

[32] Alberts I., Hünermund J.-N., Prenosil G., Mingels C., Bohn K.P., Viscione M., Sari H., Vollnberg B., Shi K., Afshar-Oromieh A. (2021). Clinical Performance of Long Axial Field of View PET/CT: A Head-to-Head Intra-Individual Comparison of the Biograph Vision Quadra with the Biograph Vision PET/CT. Eur. J. Nucl. Med. Mol. Imaging.

[33] Prenosil G.A., Sari H., Fürstner M., Afshar-Oromieh A., Shi K., Rominger A., Hentschel M. (2022). Performance Characteristics of the Biograph Vision Quadra PET/CT System with a Long Axial Field of View Using the NEMA NU 2-2018 Standard. J. Nucl. Med. Off. Publ. Soc. Nucl. Med..

[34] Nardo L., Pantel A.R. (2021). Oncologic Applications of Long Axial Field-of-View PET/Computed Tomography. PET Clin..

[35] Nardo L., Abdelhafez Y.G., Spencer B.A., Badawi R.D. (2021). Clinical Implementation of Total-Body PET/CT at University of California, Davis. PET Clin..

[36] Chondronikola M., Sarkar S. (2021). Total-Body PET Imaging. PET Clin..

[37] Henrich T.J., Jones T., Beckford-Vera D., Price P.M., VanBrocklin H.F. (2021). Total-Body PET Imaging in Infectious Diseases. PET Clin..

[38] Saboury B., Morris M.A., Farhadi F., Nikpanah M., Werner T.J., Jones E.C., Alavi A. (2020). Reinventing Molecular Imaging with Total-Body PET, Part I: Technical Revolution in Evolution. PET Clin..

[39] NCCN Guidelines. https://www.nccn.org/guidelines/category_1.

[40] Bhatla N., Berek J.S., Cuello Fredes M., Denny L.A., Grenman S., Karunaratne K., Kehoe S.T., Konishi I., Olawaiye A.B., Prat J. (2019). Revised FIGO Staging for Carcinoma of the Cervix Uteri. Int. J. Gynecol. Obstet..

[41] (2023). NCCN Guidelines. Uterine Neoplasms, Version 1. https://www.nccn.org/professionals/physician_gls/pdf/uterine.pdf.

[42] (2023). NCCN Guidelines. Cervical Cancer, Version 1. https://www.nccn.org/professionals/physician_gls/pdf/cervical.pdf.

[43] (2023). NCCN Guidelines. Vulvar Cancer, Version 1. https://www.nccn.org/professionals/physician_gls/pdf/vulvar.pdf.

[44] (2023). NCCN Guidelines. Ovarian Cancer, Version 1. https://www.nccn.org/professionals/physician_gls/pdf/ovarian.pdf.

[45] Alavi A., Saboury B., Nardo L., Zhang V., Wang M., Li H., Raynor W.Y., Werner T.J., Høilund-Carlsen P.F., Revheim M.-E. (2022). Potential and Most Relevant Applications of Total Body PET/CT Imaging. Clin. Nucl. Med..

[46] van Sluis J., Borra R., Tsoumpas C., van Snick J.H., Roya M., ten Hove D., Brouwers A.H., Lammertsma A.A., Noordzij W., Dierckx R.A.J.O. (2022). Extending the Clinical Capabilities of Short- and Long-Lived Positron-Emitting Radionuclides through High Sensitivity PET/CT. Cancer Imaging.

[47] De Gaetano A.M., Calcagni M.L., Rufini V., Valentini A.L., Gui B., Giordano A., Bonomo L. (2009). Imaging of Gynecologic Malignancies with FDG PET–CT: Case Examples, Physiolocic Activity, and Pitfalls. Abdom. Imaging.

[48] Hernandez Pampaloni M., Facchetti L., Nardo L. (2016). Pitfalls in [^18^F]FDG PET Imaging in Gynecological Malignancies. Q. J. Nucl. Med. Mol. Imaging.

[49] Basu S., Kung J., Houseni M., Zhuang H., Tidmarsh G.F., Alavi A. (2009). Temporal Profile of Fluorodeoxyglucose Uptake in Malignant Lesions and Normal Organs over Extended Time Periods in Patients with Lung Carcinoma: Implications for Its Utilization in Assessing Malignant Lesions. Q. J. Nucl. Med. Mol. Imaging.

[50] Zhuang H., Pourdehnad M., Lambright E.S., Yamamoto A.J., Lanuti M., Li P., Mozley P.D., Rossman M.D., Albelda S.M., Alavi A. (2001). Dual Time Point 18F-FDG PET Imaging for Differentiating Malignant from Inflammatory Processes. J. Nucl. Med. Off. Publ. Soc. Nucl. Med..

[51] Schillaci O. (2012). Use of Dual-Point Fluorodeoxyglucose Imaging to Enhance Sensitivity and Specificity. Semin. Nucl. Med..

[52] Cheng G., Torigian D.A., Zhuang H., Alavi A. (2013). When Should We Recommend Use of Dual Time-Point and Delayed Time-Point Imaging Techniques in FDG PET?. Eur. J. Nucl. Med. Mol. Imaging.

[53] Alberts I., Prenosil G., Mingels C., Bohn K.P., Viscione M., Sari H., Rominger A., Afshar-Oromieh A. (2021). Feasibility of Late Acquisition [68 Ga] Ga-PSMA-11 PET/CT Using a Long Axial Field-of-View PET/CT Scanner for the Diagnosis of Recurrent Prostate Cancer-First Clinical Experiences. Eur. J. Nucl. Med. Mol. Imaging.

[54] Alberts I., Schepers R., Zeimpekis K., Sari H., Rominger A., Afshar-Oromieh A. (2022). Combined [68 Ga] Ga-PSMA-11 and Low-Dose 2-[18F]FDG PET/CT Using a Long-Axial Field of View Scanner for Patients Referred for [177Lu]-PSMA-Radioligand Therapy. Eur. J. Nucl. Med. Mol. Imaging.

[55] Dendl K., Koerber S.A., Tamburini K., Mori Y., Cardinale J., Haberkorn U., Giesel F.L. (2022). Advancement and Future Perspective of FAPI PET/CT In Gynecological Malignancies. Semin. Nucl. Med..

[56] Tsujikawa T., Makino A., Mori T., Tsuyoshi H., Kiyono Y., Yoshida Y., Okazawa H. (2022). PET Imaging of Estrogen Receptors for Gynecological Tumors. Clin. Nucl. Med..

[57] Sari H., Mingels C., Alberts I., Hu J., Buesser D., Shah V., Schepers R., Caluori P., Panin V., Conti M. (2022). First Results on Kinetic Modelling and Parametric Imaging of Dynamic 18F-FDG Datasets from a Long Axial FOV PET Scanner in Oncological Patients. Eur. J. Nucl. Med. Mol. Imaging.

[58] Narayanan P., Sahdev A. (2017). The Role of ^18^ F-FDG PET CT in Common Gynaecological Malignancies. Br. J. Radiol..

[59] Wang G., Nardo L., Parikh M., Abdelhafez Y.G., Li E., Spencer B.A., Qi J., Jones T., Cherry S.R., Badawi R.D. (2022). Total-Body PET Multiparametric Imaging of Cancer Using a Voxelwise Strategy of Compartmental Modeling. J. Nucl. Med. Off. Publ. Soc. Nucl. Med..

[60] Viswanath V., Sari H., Pantel A.R., Conti M., Daube-Witherspoon M.E., Mingels C., Alberts I., Eriksson L., Shi K., Rominger A. (2022). Abbreviated Scan Protocols to Capture 18F-FDG Kinetics for Long Axial FOV PET Scanners. Eur. J. Nucl. Med. Mol. Imaging.

[61] Campos N.M.F., Almeida V., Curvo Semedo L. (2022). Peritoneal Disease: Key Imaging Findings That Help in the Differential Diagnosis. Br. J. Radiol..

[62] van’t Sant I., Engbersen M.P., Bhairosing P.A., Lambregts D.M.J., Beets-Tan R.G.H., van Driel W.J., Aalbers A.G.J., Kok N.F.M., Lahaye M.J. (2020). Diagnostic Performance of Imaging for the Detection of Peritoneal Metastases: A Meta-Analysis. Eur. Radiol..

[63] De Gaetano A.M., Calcagni M.L., Rufini V., Valenza V., Giordano A., Bonomo L. (2009). Imaging of Peritoneal Carcinomatosis with FDG PET-CT: Diagnostic Patterns, Case Examples and Pitfalls. Abdom. Imaging.

[64] van Sluis J., van Snick J.H., Brouwers A.H., Noordzij W., Dierckx R.A.J.O., Borra R.J.H., Slart R.H.J.A., Lammertsma A.A., Glaudemans A.W.J.M., Boellaard R. (2022). EARL Compliance and Imaging Optimisation on the Biograph Vision Quadra PET/CT Using Phantom and Clinical Data. Eur. J. Nucl. Med. Mol. Imaging.

[65] Sachpekidis C., Pan L., Kopp-Schneider A., Weru V., Hassel J.C., Dimitrakopoulou-Strauss A. (2022). Application of the Long Axial Field-of-View PET/CT with Low-Dose [18F]FDG in Melanoma. Eur. J. Nucl. Med. Mol. Imaging.

[66] Feudo V., Collarino A., Arciuolo D., Lorusso M., Ferrandina G., Rufini V., Collarino A., Vidal-Sicart S., Olmos R.A.V. (2022). Cervical Cancer. Nuclear Medicine Manual on Gynaecological Cancers and Other Female Malignancies.

[67] Slart R.H.J.A., Tsoumpas C., Glaudemans A.W.J.M., Noordzij W., Willemsen A.T.M., Borra R.J.H., Dierckx R.A.J.O., Lammertsma A.A. (2021). Long Axial Field of View PET Scanners: A Road Map to Implementation and New Possibilities. Eur. J. Nucl. Med. Mol. Imaging.

[68] Lowry K.P., Lee J.M., Kong C.Y., McMahon P.M., Gilmore M.E., Cott Chubiz J.E., Pisano E.D., Gatsonis C., Ryan P.D., Ozanne E.M. (2012). Annual Screening Strategies in BRCA1 and BRCA2 Gene Mutation Carriers: A Comparative Effectiveness Analysis. Cancer.

[69] Hepner A., Negrini D., Hase E.A., Exman P., Testa L., Trinconi A.F., Filassi J.R., Francisco R.P.V., Zugaib M., O’Connor T.L. (2019). Cancer During Pregnancy: The Oncologist Overview. World J. Oncol..

[70] Hu Y., Zheng Z., Yu H., Wang J., Yang X., Shi H. (2023). Ultra-Low-Dose CT Reconstructed with the Artificial Intelligence Iterative Reconstruction Algorithm (AIIR) in 18F-FDG Total-Body PET/CT Examination: A Preliminary Study. EJNMMI Phys..

[71] Sari H., Teimoorisichani M., Mingels C., Alberts I., Panin V., Bharkhada D., Xue S., Prenosil G., Shi K., Conti M. (2022). Quantitative Evaluation of a Deep Learning-Based Framework to Generate Whole-Body Attenuation Maps Using LSO Background Radiation in Long Axial FOV PET Scanners. Eur. J. Nucl. Med. Mol. Imaging.

[72] Guo R., Xue S., Hu J., Sari H., Mingels C., Zeimpekis K., Prenosil G., Wang Y., Zhang Y., Viscione M. (2022). Using Domain Knowledge for Robust and Generalizable Deep Learning-Based CT-Free PET Attenuation and Scatter Correction. Nat. Commun..

[73] Abdul-Karim F.W., Kida M., Wentz W.B., Carter J.R., Sorensen K., Macfee M., Zika J., Makley J.T. (1990). Bone Metastasis from Gynecologic Carcinomas: A Clinicopathologic Study. Gynecol. Oncol..

[74] Rose P.G., Steven Piver M., Tsukada Y., Lau T. (1989). Patterns of Metastasis in Uterine Sarcoma. An Autopsy Study. Cancer.

[75] Cormio G., Rossi C., Cazzolla A., Resta L., Loverro G., Greco P., Selvaggi L. (2003). Distant Metastases in Ovarian Carcinoma. Int. J. Gynecol. Cancer.

[76] Thanapprapasr D., Nartthanarung A., Likittanasombut P., Na Ayudhya N.I., Charakorn C., Udomsubpayakul U., Subhadarbandhu T., Wilailak S. (2010). Bone Metastasis in Cervical Cancer Patients Over a 10-Year Period. Int. J. Gynecol. Cancer.

[77] Zhang Y.-Q., Hu P.-C., Wu R.-Z., Gu Y.-S., Chen S.-G., Yu H.-J., Wang X.-Q., Song J., Shi H.-C. (2020). The Image Quality, Lesion Detectability, and Acquisition Time of 18F-FDG Total-Body PET/CT in Oncological Patients. Eur. J. Nucl. Med. Mol. Imaging.

[78] Timp J.F., Braekkan S.K., Versteeg H.H., Cannegieter S.C. (2013). Epidemiology of Cancer-Associated Venous Thrombosis. Blood.

[79] Odajima S., Seki T., Kato S., Tomita K., Shoburu Y., Suzuki E., Takenaka M., Saito M., Takano H., Yamada K. (2022). Efficacy of Edoxaban for the Treatment of Gynecological Cancer-Associated Venous Thromboembolism: Analysis of Japanese Real-World Data. J. Gynecol. Oncol..

[80] O’Gorman C.A., Minnock S., Mulhall J., Gleeson N. (2022). Attention to Bone Health in Follow-up of Gynaecological Cancers in Tertiary Care. Womens Health.

[81] Calabro’ A., Abdelhafez Y.G., Triumbari E.K.A., Spencer B.A., Chen M.S., Albano D., Cassim C.R., Bertagna F., Dondi F., Cherry S.R. (2023). 18F-FDG Gallbladder Uptake: Observation from a Total-Body PET/CT Scanner. BMC Med. Imaging.

[82] Jiang W., Chalich Y., Deen M.J. (2019). Sensors for Positron Emission Tomography Applications. Sensors.

[83] Eakin C.M., Lai T., Cohen J.G. (2022). Alarming Trends and Disparities in High-Risk Endometrial Cancer. Curr. Opin. Obstet. Gynecol..

